# *MTHFR* Gene C677T Polymorphism (rs1801133) and Susceptibility to Colorectal Polyps in an Azerbaijani Population

**DOI:** 10.3390/jcm13010219

**Published:** 2023-12-30

**Authors:** Hazi Aslanov, Bayram Bayramov, Christoph Reissfelder, Shams Abdullayeva, Zeynab Mammadova, Fikrat Aliyev, Michael Keese, Javahir Hajibabazade, Vugar Yagublu

**Affiliations:** 1 Department of Surgery, Scientific Center of Surgery after academician M.A.Topchubashov, Baku AZ1122, Azerbaijan; haslanov@yahoo.com; 2 Laboratory of Human Genetics, Genetic Resources Institute of Ministry of Science and Education, Baku AZ1106, Azerbaijan; dr.bayrambayramov@gmail.com (B.B.); zeynebmamedova053@gmail.com (Z.M.); 3 Department of Natural Sciences, Western Caspian University, Baku AZ1001, Azerbaijan; 4 Department of Surgery, Universitätsmedizin Mannheim, Medical Faculty Mannheim, Heidelberg University, 68167 Mannheim, Germany; christoph.reissfelder@umm.de; 5 Department of Neurology, Westpfalz-Klinikum, 67655 Kaiserslautern, Germany; shemsabdullayeva94@gmail.com; 6 Department of Pathomorphology, Scientific Center of Surgery after academician M.A.Topchubashov, Baku AZ1122, Azerbaijan; dr.fikret67@gmail.com; 7 Department of Vascular Surgery, Theresienkrankenhaus, 68165 Mannheim, Germany; m.keese@theresienkrankenhaus.de; 8 Carver College of Medicine, University of Iowa, Bowen Science Building, 51 Newton, Road, Iowa City, IA 52242-1009, USA

**Keywords:** colon polyp, methylenetetrahydrofolate reductase gene, single-nucleotide polymorphism, DNA, PCR-RFLP

## Abstract

Background: Understanding the relationships between the methylenetetrahydrofolate reductase (*MTHFR*) gene polymorphism, colorectal polyps, and CRC risk can aid in advancing personalized medicine approaches in CRC prevention. The aim of the current study is to identify the association of C677T polymorphism of the *MTHFR* gene with the risk of colorectal polyps in the Azerbaijani population. Methods: This study included 125 patients with colon polyps and 155 healthy individuals as a control group. DNA was extracted from venous blood samples obtained from patients and healthy individuals, and the results were analyzed through polymerase chain reaction-restriction fragment length polymorphism (PCR-RFLP) and agarose gel electrophoresis. Results: Wild-type, heterozygote, and homozygous mutant were revealed within 69 (55.2%), 49 (39.2%), and 7 (5.6%) patients and within 100 (64.5%), 45 (29%), and 10 (6.5%) healthy controls, respectively. However, no significant statistical associations were observed between CT and TT genotypes, dominant (CC vs. CT + TT) and recessive (CC + CT vs. TT) models, and the mutant T allele and disease risk. There were also no significant differences between patients and controls regarding age, sex, smoking and alcohol use. Conclusion: Our research did not reveal any significant association between the *MTHFR* C677T polymorphism and susceptibility to colorectal polyps in the Azerbaijan population.

## 1. Introduction

Colorectal cancer (CRC) stands as a formidable global health challenge, accounting for high morbidity and mortality rates [1]. The majority of CRC cases are believed to arise from precancerous growths, namely adenomatous polyps or adenomas, which possess the potential to transform into malignant neoplasms over time [2]. Despite advancements in medical knowledge and technology, there remains a critical need to elucidate the underlying processes that initiate CRC development from these precancerous lesions to optimize screening strategies for early detection [3]. Therefore, understanding the genetic factors that influence CRC and colorectal adenoma (CRA) risk is crucial [4,5]. The mechanisms underlying this association must be clarified through large-scale studies that consider gene–environment interactions and diverse populations [6]. It is expected that, these findings could provide insights into how to assess risk, screen for polyps, and intervene therapeutically for those at increased risk of colorectal cancer and polyps.

Strong evidence from epidemiological, animal, and human studies indicates that the folate (vitamin B9) status modulates the risk of developing cancers in selected tissues, notably in colon [7,8]. Multiple enzymes regulate intracellular folate metabolism, including methylenetetrahydrofolate reductase (MTHFR), methionine synthase (MTR), and methionine synthase reductase (MTRR) [9,10]. Various genetic variations in these enzymes, in addition to the level of folate intake and serum folate status, can influence their activity, thereby causing aberrant DNA methylation patterns, which can contribute to the transformation of colorectal adenomas into cancers [9]. C677T polymorphism of the gene encoding for this enzyme is evidently involved in carcinogenesis. The *MTHFR* gene C677T polymorphism has been investigated for its potential association with colorectal polyp development along with an increased risk of colorectal cancer [11,12,13]. However, there is little information regarding the association between *MTHFR* C677T polymorphism and colorectal polyp risk.

An intricate relationship between folate metabolism and stepwise progression from polyps to CRC involves genetic variations such as the *MTHFR* C677T polymorphism, along with complex interactions with alcohol and smoking [9]. A comprehensive understanding of these factors can guide strategies for colon cancer prevention, early detection, and personalized interventions, ultimately contributing to improved public health outcomes [14]. The exact route to carcinoma seems to differ between hereditary CRC syndromes, but in most cases, polyps undergo carcinogenesis [15]. Vogelstein described a multi-step model of colorectal carcinogenesis where a benign adenoma or polyp progresses into a malignant carcinoma through a series of well-defined histological stages; it is called the adenoma–carcinoma sequence model [16]. This multi-step model has been influential in advancing our understanding of colorectal cancer and has led to the identification of specific genetic markers and potential targets for treatment and prevention. The manner in which folate and related gene polymorphisms modulate colorectal carcinogenesis from adenomas to cancer remains a subject of intensive investigation [17,18]. 

The *MTHFR* gene is involved in the conversion of 5,10-methylenetetrahydrofolate to 5-methyltetrahydrofolate, which acts as a methyl group donor for the conversion of homocysteine to methionine [5]. Two major single-nucleotide polymorphisms that cause amino acid changes in the *MTHFR* gene have been identified: C677T (Ala222Val, rs1801133) and A1298C (Glu429Ala, rs1801131) [19]. Of these polymorphisms, C677T alters enzyme activity, leading to increased homocysteine concentrations in the blood and impaired folate metabolism [20,21]. Folate, a critical B vitamin, is integral to DNA synthesis, repair, and methylation [22]. These processes are fundamental in maintaining genomic integrity and regulating gene expression. An imbalance in folate metabolism, caused by genetic polymorphisms, can disrupt crucial functions and dysregulate cellular pathways, potentially causing uncontrolled cell growth, reduced apoptosis, and increased tumorigenesis [8,23]. It is imperative to consider the fact that the progression from colorectal adenoma to cancer is a multifactorial process influenced by various factors. Therefore, while studying the association of *MTHFR* C677T polymorphism with colorectal adenoma and cancer pathogenesis, current studies usually include other significant factors, such as inadequate dietary folate intake and/or excessive alcohol consumption, which may have an impact on folate metabolism. 

The objective of our study was to evaluate the association of *MTHFR* C677T polymorphisms with the risk of colorectal polyps in a population-based case–control study in an Azerbaijani population.

## 2. Materials and Methods

### 2.1. Subjects

Blood samples from patients and controls were collected at the Scientific Center of Surgery in Baku between 2017 and 2020. This study included 125 individuals with colon polyps and 155 healthy individuals. The individuals in both groups were selected randomly, and received colonoscopies within the colon cancer screening program. Venous blood samples were collected in tubes with ethylenediaminetetraacetic acid (EDTA) and then transferred to the Laboratory of Human Genetics of the Institute of Genetic Resources for DNA extraction. In addition, the colonoscopy procedures were performed using Fujinon EG-590 WL4 and Olympus CV-150 videogastroscopy devices. All patients were prepared for the colonoscopy using the clinic’s standard protocol. The patients were instructed to consume liquid food products three days before the procedure. PEG-ELP (polyethylene glycol electrolyte powder) was used for bowel preparation the day before the procedure. The procedures were performed through pancolonoscopy involving cecal intubation. The polyps removed during the colonoscopies were sent to the Pathology Laboratory of at the Scientific Center of Surgery for routine histopathological examination.

### 2.2. DNA Extraction Genotyping

**Figure 1:**
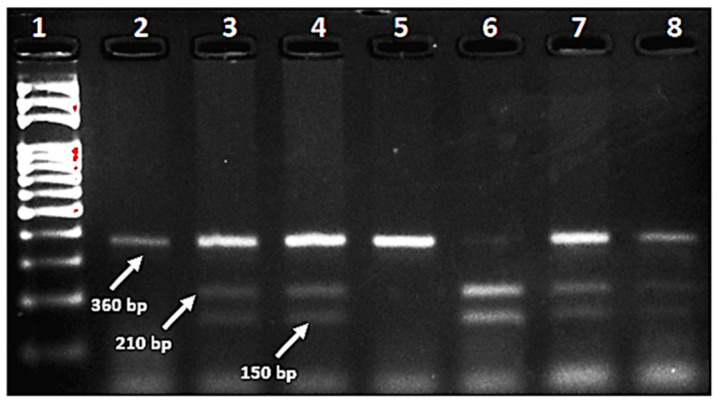
C677T genotypes of the MTHFR gene identified by the PCR-RFLP method. Lane-1: DNA Ladder (100 bp). Lane-2, 5: Wild-type CC. Lane-3, 4, 7, 8: Heterozygous CT. Lane-6: Homozygous mutant TT.

DNA extraction from blood samples was performed according to the QIAamp DNA Blood Midi Kit (QIAGEN, Hilden, Germany) protocol. DNA samples were stored at −20 °C until they were required for polymerase chain reaction (PCR) amplification. The quantity and quality of the DNA molecules were determined using a NanoDrop™ 2000/2000c spectrophotometer (ThermoFisher Scientific, Waltham, MA, USA). *MTHFR* C677T (rs1801133) genotyping was performed via polymerase chain reaction-restriction fragment length polymorphism (PCR-RFLP) methods. PCR reactions were conducted using 5′-TCCCTGTGGTCTCTTCATCC-3′ (forward) and 5′-ACTCAGCACTCCACCCAGAG-3′ (reverse) primers for *MTHFR* C677T polymorphism. The polymerase chain reaction (PCR) was conducted in a total volume of 50 μL. This included 100 ng of genomic DNA, 0.02 U of Taq DNA polymerase (obtained from Solis BioDyne, Tartu, Estonia), a 10 × Taq DNA polymerase buffer, 2 mM MgCl2, and 0.25 mM dNTP, along with 100 ng of each primer. The PCR procedure consisted of an initial denaturation step at 95 °C for 5 min, followed by 35 amplification cycles, each comprising denaturation at 95 °C for 5 min, annealing at an optimal temperature of 58 °C for 1 min, and elongation at 72 °C for 2 min. Subsequently, the PCR amplicons were visualized using a Gel Doc™ XR + Imager system (Bio-Rad, Hercules, CA, USA) on a 1% agarose gel. In a further step, the PCR products underwent processing by restriction fragment length polymorphism (RFLP) using the *HinfI* (NEB, New England Biolabs, Ipswich, MA, USA) restriction enzyme (incubated at 37 °C), and the outcomes were then analyzed on 2% agarose gel. Genotypes were visualized on agarose gel as homozygous uncut wild-type CC 360 bp, heterozygous CT consisting of 360 bp, 210 bp, and 150 bp, and homozygous mutant TT genotype 210 bp and 150 bp, respectively (Figure 1). 

### 2.3. Ethical Consideration

Our study was conducted after approval of the Ethic Committee of the Scientific Center of Surgery (Protocol number 005/08112017). This study was voluntary, and all participants provided informed written consent for the use of their blood samples and clinical information. Participants’ confidentiality and anonymity were maintained throughout this study.

### 2.4. Statistical Analysis

The analysis of the results involved biostatistical techniques using the SPSS software (version 22, SPSS, Chicago, IL, USA). The relationship between variables was assessed through Pearson’s chi-square test (χ^2^) and Fisher’s exact test. For contingency tables larger than 2 × 2, Fisher’s exact test was conducted using Social Science Statistics (http://www.socscistatistics.com/tests/chisquare2/Default2.asp (accessed on accessed on 12 March 2022)) Furthermore, a binary logistic regression was employed to compute odds ratios (ORs) along with 95% confidence intervals (CIs). All statistical tests were conducted with a two-sided approach, and significance was determined at *p* < 0.05. 

## 3. Results

### 3.1. Demographic Characteristics

The research was conducted based on blood samples taken from randomly selected 125 patients afflicted with colon polyps and 155 healthy individuals constituting the control group. Table 1 depicts the demographic and clinical data of the patients and the control subjects. Out of the total number of involved patients, 72 (57.6%) were males and 53 (42.4%) females, and there were 76 (43.6%) and 79 (56.4%) healthy male and female control subjects, respectively. Sex factors and colon polyps did not reveal a statistically significant difference (*p* = 0.153). The mean patients age was 59 ± 11.2 years (range 21–80) and 62.3 ± 13.5 years (range 28–86) in the control group. There was no statistically significant difference in age factors and colon polyps (*p* = 0.652).

### 3.2. Characteristics of Colorectal Polyps

All polyps detected during colonoscopy were histopathologically sampled. An evaluation of the histopathological findings revealed 65 tubular adenomas, 41 tubulovillous adenomas, and 19 villous adenomas. Patients with hyperplastic polyps and adenocarcinomas were excluded from this study. The localization of the polyps was as follows: 11 ascending colon, 21 transverse colon, 48 descending and sigmoid colon, 22 rectum, and 23 pancolonic. The number of polyps was categorized in the following manner: 1–5 polyps (82 patients, 65.6%), 6–10 polyps (32 patients, 25.6%), and >10 polyps (11 patients, 8.8%). The size of polyps was determined on the basis of the pathologic report and was classified into diminutive (<5 mm), small (6–10 mm), and large (>10 mm). The size of the polyps were large (>10 mm) in 36 patients (28.8%).

### 3.3. Genotype and Allele Frequencies in Patients and Controls

Outcomes concerning the *MTHFR* gene’s genotypes, allele frequencies, and the risk of colorectal polyps along with their adjusted OR and 95% Cis are summarized in Table 2. Genotypes CC, CT, and TT were revealed within 69 (55.2%), 49 (39.2%), and 7 (5.6%) afflicted patients as well as in 100 (64.5%), 45 (29%), and 10 (6.5%) healthy controls, respectively. No association was found between the incidence of heterozygous CT (OR = 1.578, 95% CI: 0.950–2.622, *p* = 0.077) and mutant homozygous TT genotype (OR = 1.014, 95% CI: 0.368–2.795, *p* = 0.978) and the risk of colorectal polyp. When it comes to the dominant (CC vs. CT + TT) (OR = 1.476, 95% CI: 0.911–2.390, *p* = 0.113) and recessive (CC + CT vs. TT) (OR = 0.860, 95% CI: 0.318–2.329, *p* = 0.767) models, no statistically significant relationships were revealed (*p* < 0.05). Wild-type C allele and mutant T allele frequencies of the *MTHFR* gene were, respectively, found to be 74.8% and 25.2% in patients and 79% and 21% in members of the control group. Although the T allele stood behind the elevated colorectal polyp risk with the OR being 1.270 and the 95% CI being 0.855–1.886, no statistical significance was pinpointed (*p* = 0.236).

The distribution of genotypes according to sex could be traced by means of Table 3. Even though the heterozygous CT (OR = 1.878, 95% CI: 0.931–3.790) and mutant TT (OR = 4.024, 95% CI: 0.404–4.101) genotypes were found to be more frequent in male patients and the situation with the wild-type CC genotype frequency was reversed by being higher in male controls, no statistically significant difference was found regarding sex and colorectal polyps (*p* = 0.077 for the CT genotype and *p* = 0.205 for the TT genotype). 

The frequency of the heterozygous CT genotype (OR = 1.350, 95% CI: 0.639–2.852) was higher in female patients, whereas the mutant TT genotype (OR = 0.714, 95% CI: 0.201–2.540) and wild-type CC genotype were observed more frequently in female control subjects. Statistical significance, however, was not found in both cases, with the *p*-value for the CT genotype being 0.431 and that for the TT genotype being 0.604.

Table 4 describes the genotype distribution with respect to age. The corresponding values of OR, 95% CI, and *p* were 2.134, 0.963–4.729, and 0.060, respectively, in the case of the heterozygous CT genotype and 0.298, 0.056–1.578, and 0.139, respectively, for the mutant TT genotype. Based on the afore mentioned outcomes, there was no statistically significant difference in age factor and colorectal polyps for the ≤59 age categories. In individuals aged over 59, the CC, CT, and TT genotypes’ distribution was 57.4%, 33.3%, and 9.3% in patients and 64.7%, 31.4%, and 3.9% in control subjects, respectively. Even though the CC genotype predominated in controls, and the CT genotype (OR = 1.198, 95% CI: 0.584–2.456) and the TT genotype (OR = 2.661, 95% CI: 0.668–10.603) were observed to be more frequent in patients, there was no statistically significant difference since the *p*-value for the CT genotype was 0.622 and that for the TT genotype was 0.268.

In addition, we analyzed the outcomes based on patients’ alcohol and tobacco consumption. In nonsmokers, both CT and mutant TT genotypes were more common than in smokers (Table 5). Furthermore, CT and TT genotypes were more prevalent among nondrinkers than among drinkers. While comparing smokers with nonsmokers (*p* > 0.05), we did not find any statistical difference between the *MTHFR* C677T polymorphism and colorectal cancer risk.

## 4. Discussion

Our study did not discover any statistically significant links between the *MTHFR* C677T polymorphism’s genotype and allele frequencies and susceptibility to colorectal adenomatous polyps. Similarly, no statistically noteworthy correlations were identified in the dominant model (CC vs. CT + TT) and the recessive model (CC + CT vs. TT). A study by Mitrou et al. in the United Kingdom on 946 polyp-free controls and 894 cases observed no overall association of *MTHFR* C677T polymorphism with adenoma risk [24]. In addition, a case–control study in Japan found no consistent connection between *MTHFR* C677T and *ALDH2* polymorphisms and colorectal adenoma [25], while the mutant 677T allele was associated with a reduced disease risk [25]. In contrast, our study found no statistical relationship between the mutant T allele and disease risk. In a population study conducted in the Netherlands, Donk et al. did not find any evidence of a connection between the *MTHFR* C677T genotype and the occurrence of colorectal adenomas [26]. In a similar vein, a meta-analysis of a large cohort of studies found no association of both C677T (4616 patients and 6338 controls) and A1298C polymorphisms (1272 patients and 1684 controls) of the *MTHFR* gene with colorectal adenoma [27]. On the other hand, Murphy et al. reported that the *MTHFR* 677 CT genotype was linked with an increased risk of adenoma recurrence [28]. In another study subjects with low plasma folate levels were compared with the CC or CT genotype. It was observed that those with the TT genotype had a reduced risk of colorectal adenoma when they had high plasma folate levels and an increased risk when they had low folate levels [29]. Ulrich et al. observed elevated risks of adenomatous polyps associated with the variant *MTHFR* genotype (*TT*) among those with low intakes of folate, vitamin B_12_, and vitamin B_6_, particularly pronounced among the elderly (>60 years of age) [30].

Studying folate metabolism in the context of adenomatous polyps as precursors of colorectal cancer is critical to understanding the mechanisms underlying the development and progression of colorectal cancer. The current evidence indicates a clear link between folate metabolism and the development of both CRA and CRC. In a meta-analysis of eleven epidemiologic studies by Park et al., low folate levels were associated with a greater prevalence or incidence of colorectal adenomas, suggesting that low folate may contribute to colorectal cancer in the early stages [31]. Numerous studies have found that folate intake was inversely associated with colorectal cancer and adenoma risk, with the highest folate intake depicting a 20–40% lower risk [32]. Observing 525,000 adults over eight years, the largest prospective cohort study found a 30% lower risk of CRC development among those consuming the greatest quantity of total folate compared with those consuming the lowest [33].

A recent meta-analysis from Sun et al. (2019), containing twenty-three case–control studies with 8339 cases and 17,731 controls, concluded that *MTHFR* rs1801131, rather than rs1801133, carries a greater risk of colorectal polyps in the UK population [34]. However, another meta-analysis from Huang et al. suggested that the *MTHFR* C677T allele depicted no association with colorectal adenoma risk, and it may provide protective effects against CRC risk in a recessive genetic model only in Asians [27]. Although recent studies have found mixed results regarding the link between the *MTHFR* C677T polymorphism and colorectal polyps, there is a need for large cohorts of patients from diverse populations [35]. 

In a population-based study, people with *MTHFR* 677CT + TT polymorphisms and low folate levels were found to be significantly more likely to develop colorectal cancer [9]. The *MTHFR* C677T allele, however, was associated with a lower risk of CRC in a previous study with an Asian population [36]. Existing studies assessing the correlation between *MTHFR* polymorphisms and the risk of CRA in different populations demonstrate inconsistent results.

It was Chen et al. who performed the first study investigating the relationship between the *MTHFR* C677T polymorphism and colorectal cancer [37]. Those researchers concluded that the *MTHFR* C677T polymorphism affected folate activity and was linked to aberrant methylation and DNA synthesis, leading to colorectal tumorigenesis. The role of folate as an important factor in maintaining genomic stability through the regulation of DNA synthesis, repair, and methylation was confirmed by various studies [38]. Therefore, further population-based studies are necessary to determine the true role of *MTHFR* polymorphisms and other modulating factors in the development of CRC and CRA.

Our study compared male and female patients with healthy individuals of the same gender based on the knowledge that gene polymorphisms associated with colon cancer risk exhibit sexual dimorphism. We found no association between C677T polymorphism and disease risk was found, when males and females were studied separately. Similarly, *MTHFR* genetic polymorphism and colorectal adenoma risk were investigated in 205 men with colorectal adenoma and 220 healthy individuals in Japan, and no correlation was reported [29]. 

Studying the role of the *MTHFR* C677T polymorphism in CRC/CRA risk requires an understanding of its interaction with folate intake, alcohol consumption, and smoking behaviors. Our study also investigated the distribution of genotypes based on factors such as alcohol consumption and smoking frequency. Alcohol consumption has long been recognized as a risk factor for colorectal cancer (CRC) [39]. Numerous studies have attempted to find a connection between alcohol consumption and CRC, mainly through two pathways [40]. In the first pathway, ethanol is oxidized into acetaldehyde, which is subsequently converted into acetate, resulting in cell exposure to a potentially carcinogenic metabolite. In the second pathway, alcohol consumption can impact folate status through multiple mechanisms, potentially leading to folate deficiency, which in turn can impact DNA synthesis and other cellular processes. Giovannucci et al. reported an association between men with the TT genotype with a high intake of alcohol and an increased risk of colorectal adenoma [41]. The US population has also demonstrated a significant interaction between the TT genotype and an increased risk of alcohol-related adenomas [42]. A highly significant association between smoking and alcohol consumption as well as the presence of colon polyps has been reported in African Americans [43]. On the contrary, our study found no association between alcohol consumption and disease risk. Additionally, in the Japanese population, *MTHFR* C677T polymorphism did not significantly affect the association between alcohol and colorectal adenoma [25].

Smoking is an independent risk factor for colon cancer [44], and it also impacts folate metabolism [45]. Smoking generates oxidative stress, depletes antioxidant reserves, and hampers folate utilization, thereby causing DNA damage and impaired DNA repair mechanisms [45]. The interaction between the *MTHFR* polymorphism and smoking in relation to colorectal polyp risk is complex and not fully understood. A few studies have explored potential interactions between the genetic variant and smoking; however, no consistent associations have been observed [46]. Alcohol and smoking synergistically amplify folate deficiency, whereas the *MTHFR* polymorphism exacerbates this vulnerability [47,48,49]. Moreover, the mortality rate is also higher among male patients [50]. In our study, we found no relationship between smoking and colorectal adenoma risk. 

Since all of the aforementioned factors have associations with CRC, it is of great importance to determine whether these factors depict the same associations with colorectal polyps. With respect to age, it is a well-established fact that senescence leads to an increased risk of various disorders, with CRC not being an exception. Multiple research groups have revealed that older people are at higher risk of developing colon cancers [51,52]; nonetheless, the incidence of CRC in younger adults has been found to be increasing [53]. In our study, there was no relationship between age of patients and *MTHFR* C677T polymorphism and CRA risk. 

Different genotypes of *MTHFR* C677T polymorphism (known as CC, CT, and TT) and factors such as smoking, alcohol consumption, age, and sex should also be considered in future investigations. In some studies, homozygous and heterozygous mutations of the *MTHFR* gene for C677T polymorphism have been reported to render the enzyme activity lower than that of the wild-type genotype [13,54].

### Limitations of This Study

Our study has a few limitations. This study can be replicated and enhanced by increasing the number of patients and controls. The samples for this study were collected from a single center. In this respect, repeating this study in a larger population may be possible by collecting samples from different centers and ethnicities, considering other polymorphisms in the *MTHFR* gene, and determining the risks. On the other hand, factors such as serum folate levels, vitamin B12, and homocysteine should be included in future correlation studies.

## 5. Conclusions

Our study, which was directed at determining the association of C677T polymorphism of the MTHFR gene with the risk of colorectal polyps in an Azerbaijani population, revealed no statistical correlations. Nonetheless, these correlations need to be validated in a larger cohort study in the future by adjusting different factors such as smoking, alcohol consumption, age, sex, and the levels of serum folate, vitamin B12, and homocysteine. 

## Figures and Tables

**Table 1:** Demographic and clinical information of patients and controls.

	Patients N = 125 (%)	Healthy Control N = 155 (%)	* p Value
Sex			
Male	72 (57.6)	76 (43.6)	0.153
Female	53 (42.4)	79 (56.4)	
Age			
Range	21–80	28–86	0.652
Mean ± SD	59 ± 11.2	62.3 ± 13.5	
Smoking			
Yes	45 (36)	61 (39.4)	0.469
No	71 (56.8)	80 (51.7)	
Unknown	9 (7.2)	14 (8.9)	
Alcohol use			
Yes	50 (40)	56 (36.1)	0.333
No	65 (52)	93 (60)	
Unknown	10 (8)	6 (3.9)	

* *p* < 0.05 was considered statistically significant.

**Table 2:** Genotype and allele frequencies of the *MTHFR* gene and the risk of colorectal polyps.

	Patients N = 125 (%)	Healthy Controls N = 155 (%)	OR (95% CI)	* p Value
Genotypes				
CC	69 (55.2)	100 (64.5)	1	-
CT	49 (39.2)	45 (29)	1.578 (0.950–2.622)	0.077
TT	7 (5.6)	10 (6.5)	1.014 (0.368–2.795)	0.978
Dominant				
CC	69 (55.2)	100 (64.5)	1	-
CT + TT	56 (44.8)	55 (35.5)	1.476 (0.911–2.390)	0.113
Recessive				
CC + CT	118 (94.4)	145 (93.5)	1	-
TT	7 (5.6)	10 (6.5)	0.860 (0.318–2.329)	0.767
Allele frequency				
C	187 (74.8)	245 (79)	1	-
T	63 (25.2)	65 (21)	1.270 (0.855–1.886)	0.236

* *p* < 0.05 was considered statistically significant.

**Table 3:** Distribution of genotypes according to sex.

Males	Patients N = 72 (%)	Healthy Controls N = 76 (%)	OR (95% CI)	* p Value
Genotypes				
CC	41 (57)	55 (72.4)	1	-
CT	28 (38.9)	20 (26.3)	1.878 (0.931–3.790)	0.077
TT	3 (4.1)	1 (1.3)	4.024 (0.404–4.101)	0.205
Females	N = 53 (%)	N = 79 (%)		
Genotypes				
CC	28 (52.8)	45 (57)	1	-
CT	21 (39.6)	25 (31.6)	1.350 (0.639–2.852)	0.431
TT	4 (7.6)	9 (11.4)	0.714 (0.201–2.540)	0.604

* *p* < 0.05 was considered statistically significant.

**Table 4:** Distribution of genotypes according to age.

Age ≤ 59	Patients N = 71 (%)	Controls N = 53 (%)	OR (95% CI)	* p Value
CC	38 (53.5)	34 (64.1)	1	-
CT	31 (43.7)	13 (24.5)	2.134 (0.963–4.729)	0.060
TT	2 (2.8)	6 (11.4)	0.298 (0.056–1.578)	0.139
Age > 59	N = 54 (%)	N = 102 (%)		
CC	31 (57.4)	66 (64.7)	1	-
CT	18 (33.3)	32 (31.4)	1.198 (0.584–2.456)	0.622
TT	5 (9.3)	4 (3.9)	2.661 (0.668–10.603)	0.268

* *p* < 0.05 was considered statistically significant.

**Table 5:** Distribution of *MTHFR* gene C677T polymorphism in smoking and alcohol consuming patients.

Genotypes	Smokers N = 45 (%)	Non-Smokers N = 71 (%)	OR (95% CI)	* p Value
CC	27 (60)	38 (53.6)	1	-
CT	16 (35.6)	28 (39.5)	0.762 (0.345–1.681)	0.500
TT	2 (4.4)	5 (6.9)	0.533 (0.096–2.961)	0.470
	Alcohol Drinkers N = 50 (%)	Non-Drinkers N = 65 (%)		
CC	30 (66.7)	34 (20)	1	-
CT	20 (44)	24 (50.8)	0.944 (0.437–2.040)	0.884
TT	0	7 (29.2)	-	-

* *p* < 0.05 was considered statistically significant.

## Data Availability

The data utilized for the research are presented and disclosed in the tables within this article.

## References

[1] Mattiuzzi C., Sanchis-Gomar F., Lippi G. (2019). Concise update on colorectal cancer epidemiology. Ann. Transl. Med..

[2] Nguyen L.H., Goel A., Chung D.C. (2020). Pathways of colorectal carcinogenesis. Gastroenterology.

[3] Zoratto F., Rossi L., Verrico M., Papa A., Basso E., Zullo A., Tomao L., Romiti A., Russo G.L., Tomao S. (2014). Focus on genetic and epigenetic events of colorectal cancer pathogenesis: Implications for molecular diagnosis. Tumor Biol..

[4] Jass J.R. (2002). Pathogenesis of colorectal cancer. Surg. Clin..

[5] Kono S., Chen K. (2005). Genetic polymorphisms of methylenetetrahydrofolate reductase and colorectal cancer and adenoma. Cancer Sci..

[6] Montazeri Z., Theodoratou E., Nyiraneza C., Timofeeva M., Chen W., Svinti V., Sivakumaran S., Gresham G., Cubitt L., Carvajal-Carmona L. (2016). Systematic meta-analyses and field synopsis of genetic association studies in colorectal adenomas. Int. J. Epidemiol..

[7] Choi S.-W., Mason J.B. (2000). Folate and carcinogenesis: An integrated scheme. J. Nutr..

[8] Sanjoaquin M.A., Allen N., Couto E., Roddam A.W., Key T.J. (2005). Folate intake and colorectal cancer risk: A meta-analytical approach. Int. J. Cancer.

[9] Panprathip P., Petmitr S., Tungtrongchitr R., Kaewkungwal J., Kwanbunjan K. (2019). Low folate status, and MTHFR 677C> T and MTR 2756A> G polymorphisms associated with colorectal cancer risk in Thais: A case-control study. Nutr. Res..

[10] Levin B.L., Varga E. (2016). MTHFR: Addressing genetic counseling dilemmas using evidence-based literature. J. Genet. Couns..

[11] Xie S.-Z., Liu Z.-Z., Yu J.-h., Liu L., Wang W., Xie D.-L., Qin J.-B. (2015). Association between the MTHFR C677T polymorphism and risk of cancer: Evidence from 446 case–control studies. Tumor Biol..

[12] Ueland P.M., Hustad S., Schneede J., Refsum H., Vollset S.E. (2001). Biological and clinical implications of the MTHFR C677T polymorphism. Trends Pharmacol. Sci..

[13] Hiraoka M., Kagawa Y. (2017). Genetic polymorphisms and folate status. Congenit. Anom..

[14] Simon K. (2016). Colorectal cancer development and advances in screening. Clin. Interv. Aging.

[15] Zauber P., Marotta S., Sabbath-Solitare M. (2017). Molecular genetic changes in benign colorectal tumors synchronous with microsatellite unstable carcinomas do not support a field defect. Int. J. Mol. Epidemiol. Genet..

[16] Vogelstein B., Fearon E.R., Hamilton S.R., Kern S.E., Preisinger A.C., Leppert M., Smits A.M., Bos J.L. (1988). Genetic alterations during colorectal-tumor development. N. Engl. J. Med..

[17] Mason J.B. (2017). Folate status and colorectal cancer risk: A 2016 update. Mol. Asp. Med..

[18] Morita M., Yin G., Yoshimitsu S.-i., Ohnaka K., Toyomura K., Kono S., Ueki T., Tanaka M., Kakeji Y., Maehara Y. (2013). Folate-related nutrients, genetic polymorphisms, and colorectal cancer risk: The fukuoka colorectal cancer study. Asian Pac. J. Cancer Prev..

[19] Teng Z., Wang L., Cai S., Yu P., Wang J., Gong J., Liu Y. (2013). The 677C> T (rs1801133) polymorphism in the MTHFR gene contributes to colorectal cancer risk: A meta-analysis based on 71 research studies. PLoS ONE.

[20] Xu L., Qin Z., Wang F., Si S., Li L., Lin P., Han X., Cai X., Yang H., Gu Y. (2017). Methylenetetrahydrofolate reductase C677T polymorphism and colorectal cancer susceptibility: A meta-analysis. Biosci. Rep..

[21] Zhao M., Li X., Xing C., Zhou B. (2013). Association of methylenetetrahydrofolate reductase C677T and A1298C polymorphisms with colorectal cancer risk: A meta-analysis. Biomed. Rep..

[22] Pieroth R., Paver S., Day S., Lammersfeld C. (2018). Folate and its impact on cancer risk. Curr. Nutr. Rep..

[23] Bailey L.B. (2003). Folate, methyl-related nutrients, alcohol, and the MTHFR 677C→ T polymorphism affect cancer risk: Intake recommendations. J. Nutr..

[24] Mitrou P.N., Watson M.A., Loktionov A.S., Cardwell C., Gunter M.J., Atkin W.S., Macklin C.P., Cecil T., Bishop T.D., Primrose J. (2006). MTHFR (C677T and A1298C) polymorphisms and risk of sporadic distal colorectal adenoma in the UK Flexible Sigmoidoscopy Screening Trial (United Kingdom). Cancer Causes Control.

[25] Hirose M., Kono S., Tabata S., Ogawa S., Yamaguchi K., Mineshita M., Hagiwara T., Yin G., Lee K.Y., Tsuji A. (2005). Genetic polymorphisms of methylenetetrahydrofolate reductase and aldehyde dehydrogenase 2, alcohol use and risk of colorectal adenomas: Self-Defense Forces Health Study. Cancer Sci..

[26] van den Donk M., Buijsse B., van den Berg S.W., Ocké M.C., Harryvan J.L., Nagengast F.M., Kok F.J., Kampman E. (2005). Dietary intake of folate and riboflavin, MTHFR C677T genotype, and colorectal adenoma risk: A Dutch case-control study. Cancer Epidemiol. Biomark. Prev..

[27] Huang Y., Han S., Li Y., Mao Y., Xie Y. (2007). Different roles of MTHFR C677T and A1298C polymorphisms in colorectal adenoma and colorectal cancer: A meta-analysis. J. Hum. Genet..

[28] Murphy G., Sansbury L.B., Cross A.J., Stolzenberg-Solomon R., Laiyemo A., Albert P.S., Wang Z., Schatzkin A., Lehman T., Kalidindi A. (2008). Folate and MTHFR: Risk of adenoma recurrence in the Polyp Prevention Trial. Cancer Causes Control.

[29] Marugame T., Tsuji E., Inoue H., Shinomiya S., Kiyohara C., Onuma K., Hamada H., Koga H., Handa K., Hayabuchi H. (2000). Methylenetetrahydrofolate reductase polymorphism and risk of colorectal adenomas. Cancer Lett..

[30] Ulrich C.M., Kampman E., Bigler J., Schwartz S.M., Chen C., Bostick R., Fosdick L., Beresford S.A., Yasui Y., Potter J.D. (2000). Lack of association between the C677T MTHFR polymorphism and colorectal hyperplastic polyps. Cancer Epidemiol. Biomark. Prev..

[31] Park Y.M., Youn J., Cho C.H., Kim S.H., Lee J.E. (2017). Circulating folate levels and colorectal adenoma: A case-control study and a meta-analysis. Nutr. Res. Pract..

[32] Kim Y.I. (2007). Folate and colorectal cancer: An evidence-based critical review. Mol. Nutr. Food Res..

[33] Gibson T.M., Weinstein S.J., Pfeiffer R.M., Hollenbeck A.R., Subar A.F., Schatzkin A., Mayne S.T., Stolzenberg-Solomon R. (2011). Pre-and postfortification intake of folate and risk of colorectal cancer in a large prospective cohort study in the United States. Am. J. Clin. Nutr..

[34] Sun M., Zhong J., Zhang L., Shi S. (2019). Genetic impact of methylenetetrahydrofolate reductase (MTHFR) polymorphism on the susceptibility to colorectal polyps: A meta-analysis. BMC Med. Genet..

[35] Kennedy D.A., Stern S.J., Matok I., Moretti M.E., Sarkar M., Adams-Webber T., Koren G. (2012). Folate intake, MTHFR polymorphisms, and the risk of colorectal cancer: A systematic review and meta-analysis. J. Cancer Epidemiol..

[36] Yang Z., Zhang X.-F., Liu H.-X., Hao Y.-S., Zhao C.-L. (2012). MTHFR C677T polymorphism and colorectal cancer risk in Asians, a meta-analysis of 21 studies. Asian Pac. J. Cancer Prev..

[37] Chen J., Giovannucci E., Kelsey K., Rimm E.B., Stampfer M.J., Colditz G.A., Spiegelman D., Willett W.C., Hunter D.J. (1996). A methylenetetrahydrofolate reductase polymorphism and the risk of colorectal cancer. Cancer Res..

[38] Duthie S.J. (2011). Folate and cancer: How DNA damage, repair and methylation impact on colon carcinogenesis. J. Inherit. Metab. Dis..

[39] Kim J., Cho Y.A., Kim D.-H., Lee B.-H., Hwang D.-Y., Jeong J., Lee H.-J., Matsuo K., Tajima K., Ahn Y.-O. (2012). Dietary intake of folate and alcohol, MTHFR C677T polymorphism, and colorectal cancer risk in Korea. Am. J. Clin. Nutr..

[40] Svensson T., Yamaji T., Budhathoki S., Hidaka A., Iwasaki M., Sawada N., Inoue M., Sasazuki S., Shimazu T., Tsugane S. (2016). Alcohol consumption, genetic variants in the alcohol-and folate metabolic pathways and colorectal cancer risk: The JPHC study. Sci. Rep..

[41] Giovannucci E., Chen J., Smith-Warner S.A., Rimm E.B., Fuchs C.S., Palomeque C., Willett W.C., Hunter D.J. (2003). Methylenetetrahydrofolate reductase, alcohol dehydrogenase, diet, and risk of colorectal adenomas. Cancer Epidemiol. Biomark. Prev..

[42] Levine A.J., Siegmund K.D., Ervin C.M., Diep A., Lee E.R., Frankl H.D., Haile R.W. (2000). The methylenetetrahydrofolate reductase 677C→ T polymorphism and distal colorectal adenoma risk. Cancer Epidemiol. Biomark. Prev..

[43] Ashktorab H., Begum R., Akhgar A., Smoot D.T., Elbedawi M., Daremipouran M., Zhao A., Momen B., Giardiello F. (2007). Folate status and risk of colorectal polyps in African Americans. Dig. Dis. Sci..

[44] Botteri E., Borroni E., Sloan E.K., Bagnardi V., Bosetti C., Peveri G., Santucci C., Specchia C., van den Brandt P., Gallus S. (2020). Smoking and colorectal cancer risk, overall and by molecular subtypes: A meta-analysis. Off. J. Am. Coll. Gastroenterol..

[45] Promthet S.S., Pientong C., Ekalaksananan T., Wiangnon S., Poomphakwaen K., Songserm N., Chopjitt P., Moore M.A., Tokudome S. (2010). Risk factors for colon cancer in Northeastern Thailand: Interaction of MTHFR codon 677 and 1298 genotypes with environmental factors. J. Epidemiol..

[46] Ulvik A., Evensen E.T., Lien E.A., Hoff G., Vollset S.E., Majak B.M., Ueland P.M. (2001). Smoking, folate and methylenetetrahydrofolate reductase status as interactive determinants of adenomatous and hyperplastic polyps of colorectum. Am. J. Med. Genet..

[47] Larsson S.C., Giovannucci E., Wolk A. (2005). A prospective study of dietary folate intake and risk of colorectal cancer: Modification by caffeine intake and cigarette smoking. Cancer Epidemiol. Biomark. Prev..

[48] Abancens M., Bustos V., Harvey H., McBryan J., Harvey B.J. (2020). Sexual dimorphism in colon cancer. Front. Oncol..

[49] Brenner H., Hoffmeister M., Arndt V., Haug U. (2007). Gender differences in colorectal cancer: Implications for age at initiation of screening. Br. J. Cancer.

[50] White A., Ironmonger L., Steele R.J., Ormiston-Smith N., Crawford C., Seims A. (2018). A review of sex-related differences in colorectal cancer incidence, screening uptake, routes to diagnosis, cancer stage and survival in the UK. BMC Cancer.

[51] Holt P.R., Kozuch P., Mewar S. (2009). Colon cancer and the elderly: From screening to treatment in management of GI disease in the elderly. Best Pract. Res. Clin. Gastroenterol..

[52] Wang T., Maden S.K., Luebeck G.E., Li C.I., Newcomb P.A., Ulrich C.M., Joo J.-H.E., Buchanan D.D., Milne R.L., Southey M.C. (2020). Dysfunctional epigenetic aging of the normal colon and colorectal cancer risk. Clin. Epigenetics.

[53] Calip G.S., Meropol N.J., Weinberg D.S. (2022). Colorectal Cancer Incidence Among Adults Younger Than 50 Years—Understanding Findings From Observational Studies of Lower Gastrointestinal Endoscopy. JAMA Oncol..

[54] Ni J., Zhang L., Zhou T., Xu W.-J., Xue J.-L., Cao N., Wang X. (2017). Association between the MTHFR C677T polymorphism, blood folate and vitamin B12 deficiency, and elevated serum total homocysteine in healthy individuals in Yunnan Province, China. J. Chin. Med. Assoc..

